# Synchronous pancreatic adenocarcinoma and duodenal mucosa‑associated lymphoid tissue lymphoma: A case report

**DOI:** 10.1097/MD.0000000000041173

**Published:** 2024-12-27

**Authors:** Yong-Pyo Lee, Jun Su Lee, Hong-Sik Kim, Hye Sook Han, Jisun Lee, Chang Gok Woo, Ok-Jun Lee, Seung-Myoung Son

**Affiliations:** a Departments of Internal Medicine, Chungbuk National University Hospital, Chungbuk National University College of Medicine, Cheongju, Republic of Korea; b Department of Radiology, Chungbuk National University Hospital, Chungbuk National University College of Medicine, Cheongju, Republic of Korea; c Department of Pathology, Chungbuk National University Hospital, Chungbuk National University College of Medicine, Cheongju, Republic of Korea.

**Keywords:** adenocarcinoma, duodenum, MALT lymphoma, pancreas, synchronous

## Abstract

**Rationale:**

Duodenal mucosa-associated lymphoid tissue (MALT) lymphoma is a rare condition. Simultaneous presence of pancreatic ductal adenocarcinoma along with duodenal MALT lymphoma has not been documented in the scientific literature. We report an exceptionally rare case of synchronous duodenal MALT lymphoma and pancreatic ductal adenocarcinoma.

**Patient concerns:**

A 75-year-old man was referred to our hospital with dyspepsia and weight loss.

**Diagnoses:**

Esophagogastroduodenoscopy was performed, revealing synchronous tumor comprising pancreatic ductal adenocarcinoma and MALT lymphoma of the duodenum.

**Interventions:**

Given that the pancreatic carcinoma would be the primary determinant of prognosis, we prioritized treatment of the pancreatic carcinoma. Consequently, we performed a Whipple operation first. Post-operative pathologic examination revealed metastasis of pancreatic cancer to peri-pancreatic lymph nodes, whereas the MALT lymphoma was localized to the duodenum; therefore, only adjuvant chemotherapy for pancreatic cancer was performed.

**Outcomes:**

To date, the patient has had no recurrence of either the pancreatic cancer or the MALT lymphoma.

**Lessons:**

To the best of our knowledge, this is the first case to be reported. Awareness of this co-occurrence may help diagnosis and management of similar cases.

## 1. Introduction

Mucosa-associated lymphoid tissue (MALT) lymphoma, first recognized as a distinct clinicopathological entity in the early 1980s, is defined as an extranodal lymphoma comprising heterogeneous B cells, which develops most frequently from the gastric mucosa.^[1,2]^ Duodenal MALT lymphoma is particularly rare, comprising only about 0.9% of all gastrointestinal malignant lymphomas.^[3]^ Simultaneous coexistence of a pancreatic ductal adenocarcinoma associated with a duodenal MALT lymphoma has not been described previously in the scientific literature. Here, we report a 75-year-old man with synchronous duodenal MALT lymphoma and pancreatic ductal adenocarcinoma.

## 2. Case presentation

The Institutional Review Board of Chungbuk National University Hospital approved the study (IRB No: 2024-06-001), and this study was performed with written informed consent and adhered to the guidelines established by the Declaration of Helsinki.

**Figure 1.**
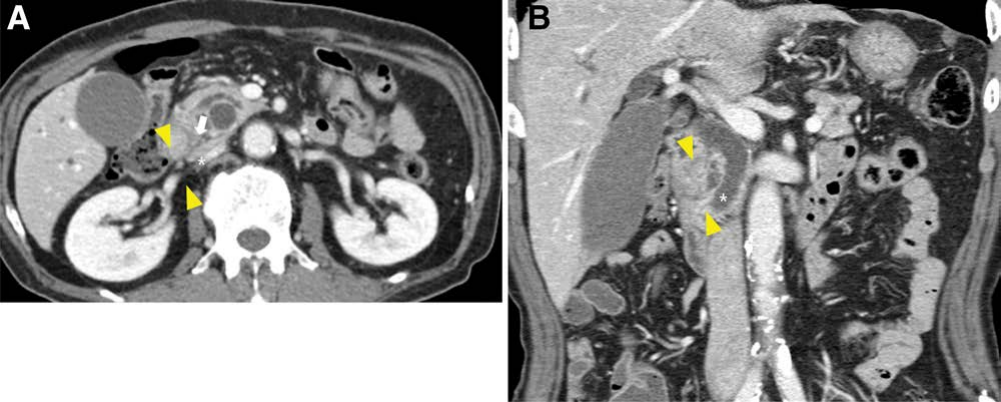
An ill-defined low-density lesion (arrowheads), with a small cystic portion in the pancreatic head, is suggestive pancreatic cancer. This is accompanied by a double-duct sign dilatation of the main pancreatic duct (arrow) and the common bile duct (asterisk), as observed on axial (A) and coronal (B) CT images. CT = computed tomography.

**Figure 2.**
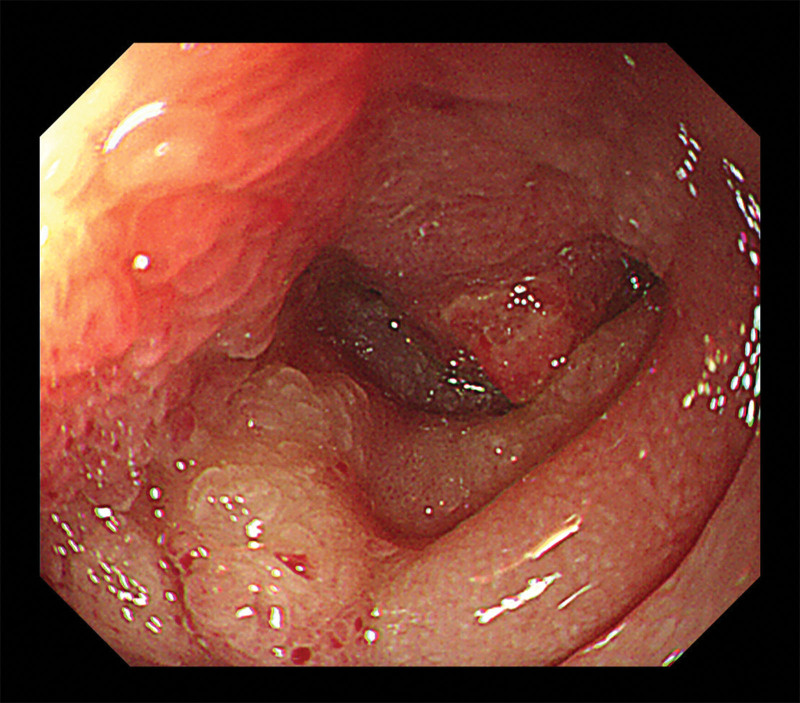
Esophagogastroduodenoscopy shows a multi-nodular mucosal lesion in the proximal second portion of the duodenum.

**Figure 3.**
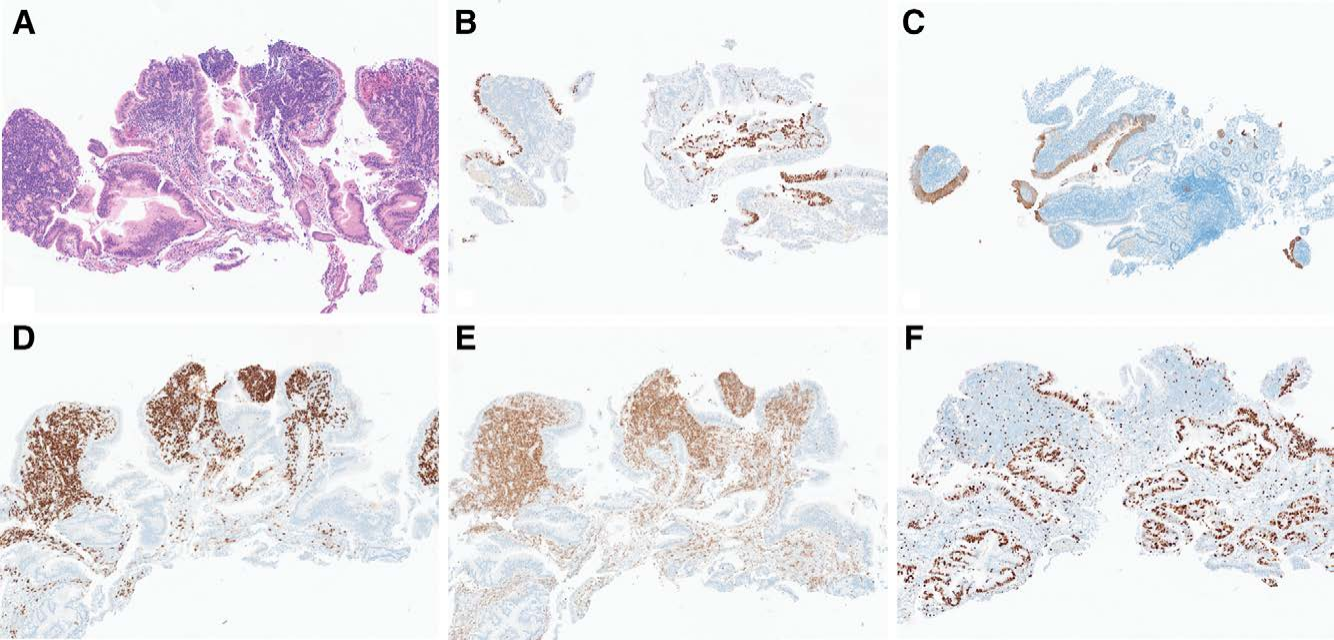
(A) Histological findings from duodenal biopsy specimens. The glandular portion was diagnosed as adenocarcinoma, which was positive for p53 (B) and CK7 (C). The lamina propria was diffusely infiltrated by atypical small lymphoid cells, which were positive for CD20 (D) and BCL2 (E). The Ki67-labeling index was high in the glandular portion and low in the lymphoid cells (F) (magnification, all ×100).

In October 2023, a 75-year-old man was referred to our hospital with dyspepsia and weight loss. He had no past medical history. The patient presented to a local hospital for assessment and underwent esophagogastroduodenoscopy; however, a tissue biopsy revealed only superficial mucosa, with lymphoid infiltration of the duodenal mucosa. Subsequent abdominal computed tomography (CT) revealed an ill-defined low-density lesion with a small cystic portion in the pancreatic head, suggestive of pancreatic cancer; this finding was accompanied by a double-duct sign dilatation of the main pancreatic duct and the common bile duct (Fig. 1A, B). Laboratory tests revealed elevated CA19-9 (103 U/mL; normal range, <37 U/mL) and normal CEA (2.11 ng/mL; normal range, <5 ng/mL) levels. Therefore, esophagogastroduodenoscopy was performed again at our hospital, revealing a multi-nodular mucosal lesion in the proximal second portion of the duodenum (Fig. 2). Duodenal biopsy specimens showed diffuse infiltration of the duodenal lamina propria by small atypical lymphoid cells, along with an atypical glandular lesion. Immunohistochemical staining revealed that the atypical glands were strongly positive for p53 and CK7, with a Ki-67 labeling index >80%. The lymphoid cells were positive for CD20 and BCL2, focal-positive for CD3, and negative for CD5, CD10, Bcl-6, and Cyclin D1, with a Ki-67 labeling index <5% (Fig. 3A–F). Furthermore, an IgH gene rearrangement test confirmed proliferation of monoclonal B-cells.

Preoperatively, we presumed that the lesion was a synchronous tumor comprising pancreatic ductal adenocarcinoma and MALT lymphoma of the duodenum. Additionally, PET-CT revealed focal uptake of FDG only in the second portion of the duodenum, with no lymph node enlargement observed on chest and abdominal CT. Thus, the duodenal MALT lymphoma was classified as stage i.e. according to the Ann Arbor staging classification. Given that the pancreatic carcinoma would be the primary determinant of prognosis, we prioritized treatment of the pancreatic carcinoma. We planned to perform a Whipple’s operation first, and then consider carefully whether to treat the MALT lymphoma once the patient had recovered from the operation.

**Figure 4.**
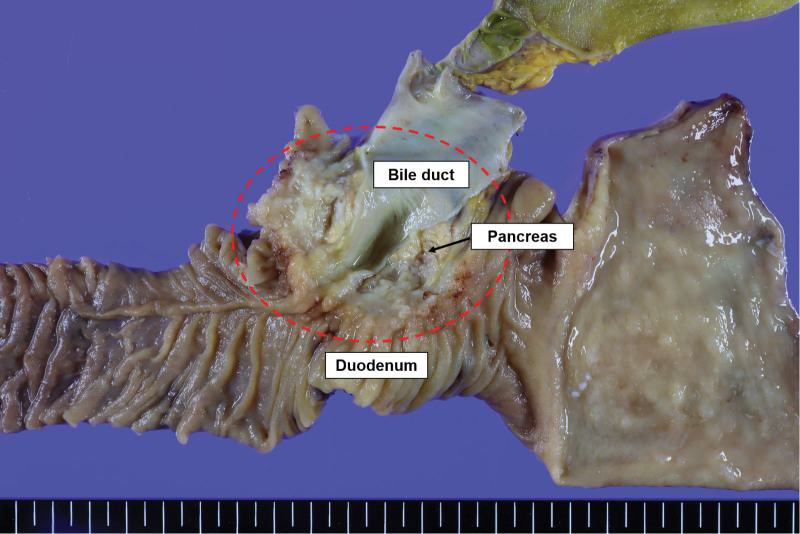
Gross examination of the surgically resected specimen revealed an ill-defined, grayish-white firm mass located in the head of the pancreas. The second portion of the duodenum, located just above the pancreatic cancer, exhibited multiple nodular lesions in the mucosal layer. The red dashed circle indicates the mass.

**Figure 5.**
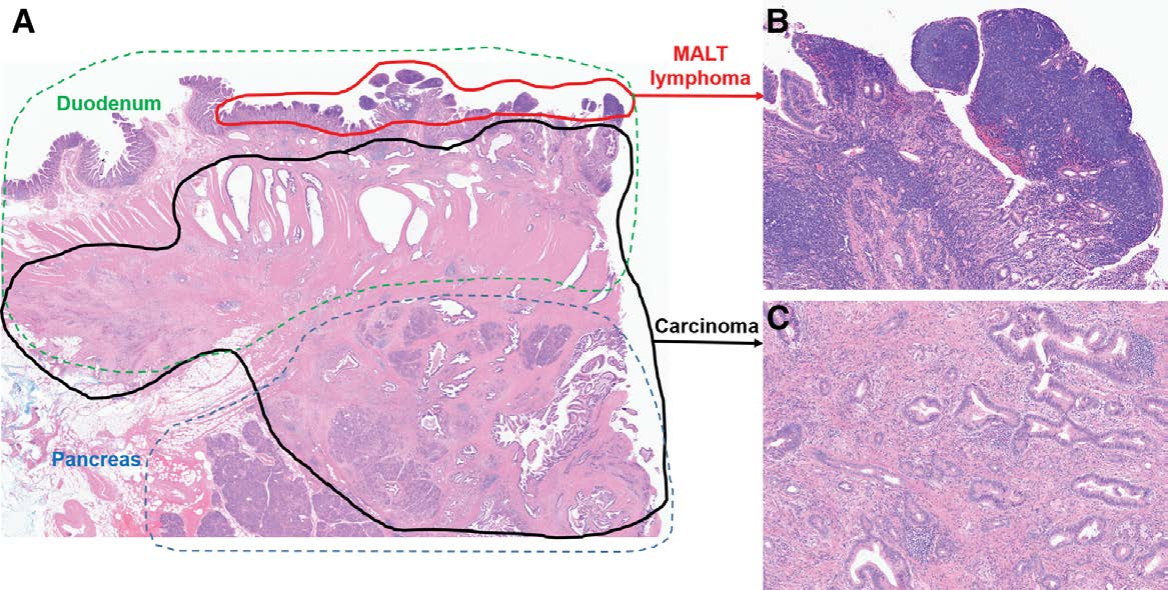
(A) The mass comprised 2 different tumors (magnification, ×10). (B) Diffuse proliferation of small lymphoid cells was observed within the duodenal mucosa adjacent to the pancreatic carcinoma (magnification, ×100). (C) The major component was well-differentiated atypical glands, a finding compatible with a diagnosis of pancreatic ductal adenocarcinoma (magnification, ×100). The borders of each tumor component are marked in red and black, respectively.

**Figure 6.**
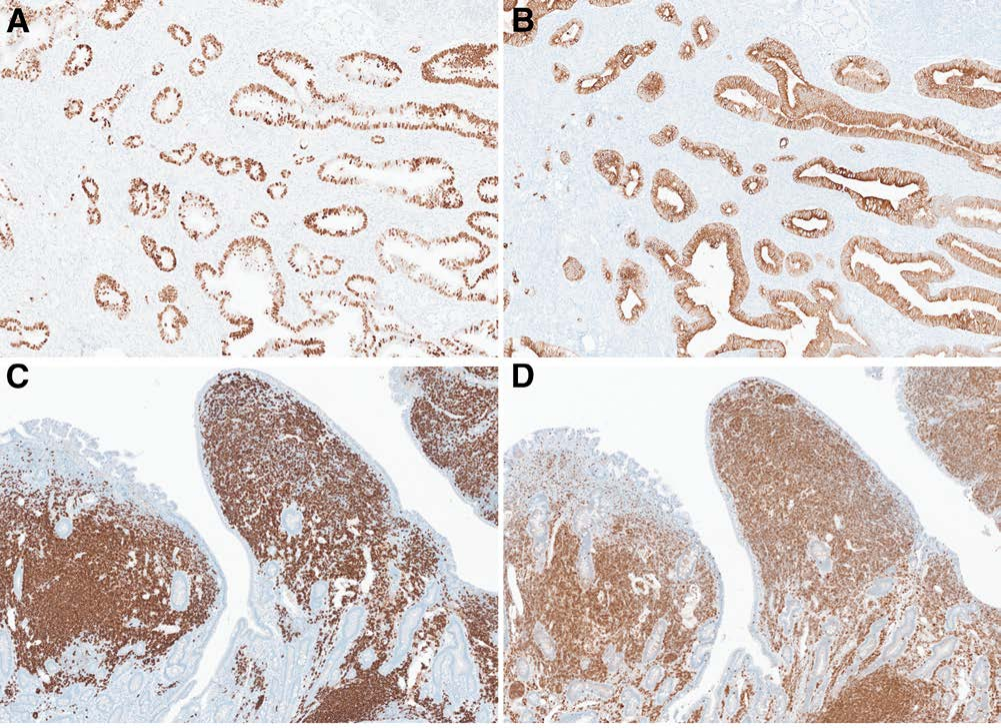
Immunohistochemical staining revealed that the adenocarcinoma component was positive for p53 (A) and CK7 (B). The MALT lymphoma component was diffusely positive for CD20 (C) and BCL2 (D) (magnification, all × 400). MALT = mucosa-associated lymphoid tissue.

Gross examination revealed an ill-defined, grayish-white firm mass measuring 5.5 × 3 × 2.5 cm in size, located in the head of the pancreas; this was considered to be pancreatic carcinoma. The second portion of the duodenum, located just above the pancreatic cancer, exhibited multiple nodular lesions in the mucosal layer (Fig. 4). Microscopically, the tumor comprised well-differentiated atypical glands invading the common bile duct and duodenum, a finding compatible with the diagnosis of pancreatic ductal adenocarcinoma. The duodenal mucosa adjacent to the pancreatic carcinoma showed diffuse proliferation of small lymphoid cells (Fig. 5A–C). Immunohistochemical staining revealed that the pancreatic ductal adenocarcinoma cells were diffusely positive for p53 and CK7. The infiltrating lymphoid cells were diffusely positive for CD20 and BCL2, and negative for CD3 (Fig. 6A–D). Lymphoepithelial lesions were rarely observed. The duodenal lesion was diagnosed as extranodal marginal zone lymphoma of mucosa-associated lymphoid tissue (i.e., MALT lymphoma). Additionally, 2 regional lymph node metastases arising from the primary pancreatic ductal adenocarcinoma were identified, resulting in a final staging of pT3pN1M0, pStage IIB according to the AJCC 8th edition criteria. Therefore, the final diagnosis was a synchronous tumor comprising pancreatic ductal adenocarcinoma and duodenal MALT lymphoma.

Imaging studies confirmed that the MALT lymphoma did not involve other organs, thereby classifying it as Ann Arbor stage IE. Complete removal of the lymphoma was achieved through Whipple’s operation, obviating the need for additional treatment. Consequently, adjuvant chemotherapy was planned solely for the pancreatic cancer. At 2 months postsurgery, the patient received 3 months of post-operative chemotherapy comprising 6 cycles of FOLFIRINOX (fluorouracil + leucovorin + irinotecan + oxaliplatin). The patient was still alive and without recurrence at the 6 month follow-up.

## 3. Discussion

Primary MALT lymphoma accounts for approximately 8% of all non-Hodgkin lymphomas. The stomach is the most common site, comprising about one-third of cases.^[4]^ MALT lymphoma is observed rarely in the duodenum.^[3]^ Its concurrent occurrence with pancreatic adenocarcinoma is exceptionally rare, and represents a diagnostic surprise. We believe that this case report is the first to describe a synchronous tumor comprising pancreatic ductal adenocarcinoma and duodenal MALT lymphoma. Notably, there has been only 1 previous case report of a collision tumor comprising pancreatic ductal adenocarcinoma and MALT lymphoma occurring in the pancreas.^[5]^

Due to its rarity, information about the clinical presentation, natural history, and treatment of duodenal lymphoma is limited. According to some case reports, symptoms range from asymptomatic to atypical abdominal pain, melena, gastric discharge, and gastric outlet obstruction.^[6–9]^ No endoscopic findings relate specifically to duodenal MALT lymphoma. MALT lymphoma of the small intestine may present with various endoscopic findings, including loss of circular folds, a cobblestone or nodular appearance, multiple sessile polyps, ulcers, and a diffuse infiltrative pattern. Similar endoscopic characteristics can be observed in cases of duodenal MALT lymphoma.^[6]^ Considering the etiology of MALT lymphoma, there is a hypothesis that duodenal MALT lymphoma may also be caused by Helicobacter pylori (H. pylori) infection of the ectopic gastric mucosa; however, duodenal MALT lymphoma is often reported without any evidence of H. pylori infection in biopsy specimens. The rate of H. pylori infection in cases of duodenal MALT lymphomas is 46.2%, compared with 90.8% in cases of gastric MALT lymphoma^[10]^; therefore, the relationship between duodenal MALT lymphoma and H. pylori infection remains unclear.^[11]^ Notably, a case report has documented that H. pylori infection confirmed in the stomach and duodenal MALT lymphoma completely remitted after H. pylori eradication. This suggests a potential intrinsic link between H. pylori infection and the development of duodenal MALT lymphoma.^[11]^

Several hypotheses have been proposed to explain the etiology of collision tumors. One theory suggests that 2 primary tumors may arise independently but happen to coexist due to a coincidental “meeting.” Alternatively, distinct tumors might develop in close proximity due to the influence of shared carcinogenic stimuli.^[12,13]^ For instance, in cases of gastric collision tumors comprising gastric cancer and MALT lymphoma, this theory could apply, as persistent H. pylori infection has been identified as a potential risk factor for both neoplasms.^[14]^ However, this explanation does not align with our case. Another possibility is that the first tumor modifies the local microenvironment, facilitating the development of a second tumor nearby.^[13]^ Specifically, MALT lymphoma could, by compromising the individual’s immune system, promote the malignant transformation of a preexisting precancerous lesion.

There is no definitive optimal treatment for duodenal MALT lymphomas. Treatment options for non-gastric MALT lymphoma include surgery, radiotherapy, rituximab (alone or in combination with chemotherapy), and observation.^[15]^ While gastric MALT lymphoma is often associated with H. pylori infection, and can often go into complete remission after eradication of H. pylori, this is not the case for duodenal MALT lymphoma. The overall complete remission rate and remission rate following initial H. pylori eradication therapy are significantly lower in cases of duodenal MALT lymphoma than in cases of gastric MALT lymphoma^[10]^; thus, treatment outcomes for duodenal MALT lymphoma appear to be inferior to those for gastric MALT lymphoma. In general, however, non-gastric MALT lymphoma has a good prognosis, with favorable survival rate: the 5-year overall survival rate is 90% and the 10-year overall survival rate is 80%, regardless of the treatment selected.^[16,17]^ Local treatment by radiotherapy or surgical resection is the mainstay for limited-stage MALT lymphoma, and if the MALT lymphoma is resected in conjunction with diagnosis, observation is an option if no residual lymphoma remains.^[18]^

In the case of synchronous tumors (as in the present case), the therapeutic strategy (including the treatment priority for each disease) is important. In this case, the MALT lymphoma was located in the second portion of the duodenum abutting the pancreatic cancer, and was within the operative field; therefore, Whipple’s operation was prioritized. Post-operative pathologic examination revealed metastasis of pancreatic cancer to peri-pancreatic lymph nodes, whereas the MALT lymphoma was localized to the duodenum; therefore, only adjuvant chemotherapy for pancreatic cancer was performed and, to date, the patient has had no recurrence of either the pancreatic cancer or the MALT lymphoma.

## 4. Conclusions

This is the first report of a surgically resected synchronous tumor comprising pancreatic ductal adenocarcinoma and duodenal MALT lymphoma. Pathologists and physicians must be vigilant for this condition. Treatment should be based on the tumor stage of the adenocarcinoma, as well as on the grade of lymphoma, and any therapeutic decision should be made with careful consideration of these factors.

## Acknowledgment

The authors would like to thank Bioedit (https://www.bioedit.com/) for the English language review.

## Author contributions

**Conceptualization:** Seung-Myoung Son.

**Data curation:** Jun Su Lee, Hong-Sik Kim, Hye Sook Han.

**Investigation:** Jun Su Lee, Hong-Sik Kim, Chang Gok Woo.

**Resources:** Jisun Lee, Chang Gok Woo, Ok-Jun Lee.

**Supervision:** Ok-Jun Lee.

**Writing – original draft:** Yong-Pyo Lee, Seung-Myoung Son.

**Writing – review & editing:** Hye Sook Han, Ok-Jun Lee.
